# Antidiarrheal activity of extracts and compound from *Trilepisium madagascariense* stem bark

**DOI:** 10.4103/0253-7613.66839

**Published:** 2010-06

**Authors:** Gerald Ngo Teke, Jules-Roger Kuiate, Victor Kueté, Rémy Bertrand Teponno, Léon Azefack Tapondjou, Gerard Vilarem

**Affiliations:** Laboratory of Microbiology and Antimicrobial Substances, Faculty of Sciences, University of Dschang, PO Box 67 Dschang, Cameroon; 1 Department of Chemistry, Faculty of Science, University of Dschang, Box 183, Dschang, Cameroon; 2 Laboratoire de Chimie Agro-Industrielle - UMR 1010 INRA/INP-ENSIACET 4, Allée Emile Monso 31432 Toulouse Cedex 4, France

**Keywords:** Antidiarrheal, castor oil, isoliquiritigenin, Shigella, *Trilepisium madagascariense*

## Abstract

**Objective:**

The present study was performed to evaluate the preventive and curative antidiarrheal effects of the methanol extract, fractions and compound from the stem bark of *Trilepisium madagascariense* in rats.

**Materials and Methods:**

The methanol extract from the stem bark of *T. madagascariense*, its fractions (n-hexane, ethyl acetate, n-butanol and aqueous residue) and compound (obtained from further column chromatography of the ethyl acetate fraction) were evaluated for the antidiarrheal activity in rats. These test samples (at 100, 200 and 400 mg/kg for the extract and fractions and 2.5 mg/kg for compound) were assayed on the latent periods, purging indices and fecal frequencies in castor oil-induced diarrhea. Gastrointestinal transit and castor oil-induced enteropooling assays were conducted. Shigella-induced diarrhea was assayed. Blood chemistry and fecal Shigella load were examined.

**Results:**

The fractionation of the ethyl acetate fraction from the methanol extract of *T. madagascariense* afforded a known compound [isoliquiritigenin (1)]. Compound 1 increased the latent period of diarrhea induction (179.40 min) compared to the saline control (60.80 min). The purging indices, fecal frequencies and intestinal enteropooling decreased with an increase in the dose of test samples. The blood cell counts, sera creatinine and fecal Shigella load decreased significantly (*P* ≤ 0.05) in the plant extract-treated rats compared to the saline control.

**Conclusion:**

The results of our study, being reported for the first time, provide clear evidence that the methanol extract, fractions and isoliquiritigenin from *T. madagascariense* stem bark possess antidiarrheal activities.

## Introduction

Diarrhea is characterized by an increase in the frequency of bowel movements, wet stool and abdominal pains.[1] It is the world’s third highest killer disease, contributing substantially to pediatric morbidity and mortality, especially in the malnourished.[2,3] The incidence of diarrhea is still high (about 7.1 million per year), despite the efforts of international organizations to control this disease.[4] Antibiotics used as antidiarrheal drugs sometimes provoke adverse effects and microorganisms tend to develop resistance toward them.[5] Therefore, the search for safe and more effective agents from plant origin has continued to be an important area of active research.

*Trilepisium madagascariense* DC, Leeuwenberg (Moraceae) is a forest tree that grows to a height of about 30 m and is usually found in riverine ground lowlands and submountaineous forest in Tropical Africa and Madagascar. It is propagated by bud grafting.[6,7] The stem bark is traditionally used to treat venereal diseases, arthritis, rheumatism, diarrhea and dysentery while the roots are used against cutaneous and subcutaneous parasitic infections.[8] The methanol extract from the leaves of *T. madagascariense* was reported to inhibit the growth of *Staphylococcus aureus*.[9]

There is, however, little scientific information regarding its general pharmacological activity. The present study was undertaken to evaluate the antidiarrheal activity of the methanol extract, fractions and compound from the stem bark of this plant in both castor oil- and Shigella-induced diarrhea models in rats.

## Materials and Methods

### Drugs, Chemicals and Kits

The following drugs, chemicals and kits were used in the study: loperamide (Sigma Aldrich; Steinheim, Germany), diphenoxylate HCl (Sigma Aldrich), atropine sulfate (Sigma Aldrich), vegetable charcoal (Carbophos^®^, AJC Pharma, Angoulem, France) and castor oil from a local pharmacy, chloroform (BDH Chemicals Ltd., Poole, England), creatinine kit (Jeffe-Kinetic, Germany) and triglycerides kit (IVD, SGM Italia-Roma), Tween 80 (Fisher Scientific, Loughborough, United Kingdom) and SS agar (Liofilchem).

### Extract Preparation

The stem bark of *T. madagascariense* was harvested in May 2007 in the lowlands around the campus of the University of Dschang in Menoua Division, West Cameroon. Its identification was established by the National Herbarium in Yaoundé-Cameroon (ref: no.9511/44014/HNC), where a voucher specimen was kept for further references. The stem bark was cut into pieces and air-dried under shade. The powdered stem bark (3 kg) was repeatedly macerated (three times) with 8 L of methanol for 24 h and the solvent was evaporated from the extract under reduced pressure in a rotary evaporator at 45°C to afford the methanol extract (340.8 g) with a yield of 11.36% w/w. A quantity of 128 g of this extract was predissolved in 200 ml of a mixture of methanol and water (1:9) and then shaken vigorously in 500 ml of *n*-hexane. The *n*-hexane phase was collected and the process was repeated thrice. Methanol was then evaporated from the polar phase and the residue was fractionated with ethyl acetate and, finally, with *n*-butanol. The aqueous residue that resulted was kept in the oven at 40°C for 48 h to evaporate the residual water. The *n*-hexane, ethyl acetate and *n*-butanol were separately evaporated under reduced pressure in a rotary evaporator to afford the *n*-hexane (10.70 g), ethyl acetate (51.80 g), *n*-butanol (9.10 g) and aqueous residue (56.4 g) fractions. The methanol extract and fractions were subjected to phytochemical screening for the identification of major groups of chemical constituents using standard procedures.[10]

### Fractionation and Isolation

The ethyl acetate fraction was subjected to column chromatography. Forty-five grams of this fraction were adsorbed onto silica gel and applied to a neutral silica gel 60 (0.063–0.200 mm) column (60 cm × 8 cm). The column was eluted with mixtures of *n*-hexane (Hex) and ethyl acetate (EtOAc) of increasing polarity (100:0, 90:10, 80:20, 70:30, 60:40, 50:50, 40:60, 30:70, 20:80 and 0:100) to give 10 fractions (F1 to F10), of which F1 was oily. Fraction F7 was further purified on a sephadex gel column using Hex-EtOAc (30:70) as the elution system. A total of 67 column fractions (10 ml each) were collected and grouped into four sephadex fractions, denoted F7.1 (1–16), F7.2 (17–22), F7.3 (23–36) and F7.4 (37–67), based on their thi*n*-layer chromatograms. Fractions F7.1, F7.2 and F7.3 were of negligible quantities (2.00 mg, too small for the performance of biological assays). Fraction F7.4 was further subjected to a silica gel column chromatography and eluted with Hex-EtOAc (30:70). A total of 28 column fractions (10 ml each) were collected and grouped into four subfractions, denoted F7.4.1 (1–15), F7.4.2 (16–18), F7.4.3 (19–25) and F7.4.4 (26–28). Fraction F7.4.1 was pure (compound 1, 45.1 mg) while F7.4.2–F7.4.4 were of negligible quantities.

### General Procedure

An aluminum sheet precoated with silica gel 60 GF _254_ nm (Merck, Darmstadt, Germany) was used for thi*n*-layer chromatography (TLC). The spots were visualized under ultraviolet (UV) light (254 and 366 nm), UV lamp model 52-58 mineralight, and sprayed with 50% aqueous solution of H_2_SO_4_ followed by heating at 100°C.

Infrared spectra were measured as a film on a KBr pellet using the FT-IR-8400S Shimadzu spectrometer. Electron impact mass spectrometry (EI-MS) were carried out on a GCT Premier CAB109 TOF mass spectrometer. ^1^H-, ^13^C-NMRand 2D-NMR (COSY, HMBC and HSQC) spectra were recorded in acetone-d_6_ (500 MHz for ^1^H and 125 MHz for ^13^C) on a Bucker 500 MHz NMR instrument. All chemical shifts (*δ*) were given in ppm units with reference to tetramethylsilane as an internal standard and coupling constants (*J*) were in Hz.

Isoliquiritigenin: yellow amorphous powder; IR λ_max_ (KBr, cm^-1^): 3500, 1630, 1370; ^1^H-NMR (acetone-d _6_): *δ* 8.15 (1H, d, *J* = 8.0 Hz, H-6’), 7.82 (1H, d, *J* = 15.2 Hz, H-*β*), 7.79 (1H, d, *J* = 15.2 Hz, H-*α*), 7.76 (2H, d, *J* = 8.2, H-2, H-6), 6.95 (2H, d, *J* = 8.2 Hz, H-3, H-5), 6.48 (1H, *dd, J* = 8.0, 2.0 Hz, H-5’), 6.39 (1H, *d, J* = 2.0 Hz, H-3’); ^13^C-NMR (acetone-d_6_): *δ* 192.0 (CO), 166.7 (C-4’), 164.6 (C-2’), 160.1 (C-4), 144.3 (C-*β*), 132.5 (C-6’), 130.9 (C-2, C-6), 126.7 (C-1), 117.6 (C-*α*), 115.9 (C-3, C-5), 113.7 (C-1’), 107.7 (C-5’), 102.8 (C-3’); EI-MS *m/z* (%relative intensity): 256 (M^+^, 100), 255 (60), 239 (17), 163 (26), 137 (62), 120 (30).

### Animals Used

Wistar albino rats (170–180 g), both males and females, were used for the antidiarrheal test. They were housed in standard plastic cages and provided with food and water *ad libitum*. The studies were conducted according to the ethical guidelines of the Committee for Control and Supervision of Experiments on Animals (Registration no. 173/CPCSEA, dated 28 January, 2000), Government of India, on the use of animals for scientific research.

### Castor Oil-Induced Diarrhea in Rats

The animals were starved for 18 h prior to the experiments and were randomly distributed into nine groups of 10 (five males and five females) animals per study dose of the test substances. Rats of the first three groups were administered orally by gavage 100, 200 and 400 mg/kg body weight of the methanol extract, respectively. Groups 4, 5, 6 and 7 received 80 mg/kg of the *n*-hexane, ethyl acetate, *n*-butanol and aqueous residue fractions, respectively. Groups 8 and 9 (positive controls) received loperamide (Imodium) and diphenoxylate HCl, respectively, at 2.5 mg/kg body weight as reference drugs while group 10 animals were given compound 1 (2.5 mg/kg). The eleventh group (negative control) received 5% ethanol/tween 80 in normal saline (1 ml/100 g body weight). Sixty minutes after drug treatment, each animal was administered castor oil orally (1 ml/100 g body weight). The latent period (the time between castor oil administration and appearance of first diarrheic drop) was recorded. Observation for defecation continued up to 6 h on filter paper placed beneath the individual perforated rat cages. This paper was replaced every hour after noting its weight (M_1_). Finally, the filter paper was exposed in the laboratory for drying over a period of 14 h and it was reweighed (M_2_). The fecal water content was calculated as (M_2_ – M_1_) g. The percentage of rats that responded to diarrhea, the latent period, the mean stool frequency, frequency of diarrheic drops and water content were recorded.[11] The purging indices,[12] the percentage inhibition of defecation and diarrheic drops[1] were evaluated.

$$\mathrm{Purging}\;\mathrm{index}\;=\;\frac{\%\;\mathrm{respondants}\;\times \;\mathrm{average}\;\mathrm{number}\;\mathrm{of}\;\mathrm{stool}}{\mathrm{Average}\;\mathrm{latent}\;\mathrm{period}}$$

$$\mathrm{Inhibition}\;\mathrm{of}\;\mathrm{defecation}\;(\%)\;=\;\frac{\mathrm{Mc}\;-\;\mathrm{Md}}{\mathrm{Mc}}\;\times \;100$$

$$\mathrm{Inhibition}\;\mathrm{of}\;\mathrm{diarrhoeic}\;\mathrm{drops}\;=\;\frac{\mathrm{Mo}\;-\;\mathrm{Me}}{\mathrm{Mo}}\;\times \;100$$

### Effects of Plant Extracts on Castor Oil-Induced Enteropooling

Enteropooling (intraluminal fluid accumulation) was determined as described by Murugesan *et al*.[11] For this evaluation, rats deprived of food and water for 18 h were randomly selected into six groups and were placed in plastic cages. The first three groups received the methanol extract at 100, 200 and 400 mg/kg, respectively. The fourth and fifth groups (positive controls) received loperamide and diphenolate HCl (both at 2.5 mg/kg body weight) as standard drugs while the sixth group (negative control) received 5% ethanol/tween 80 in normal saline at 1 ml/100 g body weight. Thirty minutes after drug treatment, castor oil was administered to all the rats (1 ml/100 g body weight). Thirty minutes after castor oil administration, each rat was sacrificed and the ends of the small intestine were tied (at both the pylorus and the caecum). This section was dissected out and its length was measured. The intestinal content was collected by milking into preweighed (m_0_) graduated tubes and the new weight (m_1_) was measured. The volume (ml) of the intestinal content was read directly from the graduation while the mass was obtained as (m_1_ -m_0_) g.

### Effects of Plant Extracts on Gastrointestinal Motility

The method described by Akah *et al*.[13] was used. Another set of rats were fasted for 18 h, but they had free access to water. The animals of the first three test groups (*n* = 8) received the methanol extract orally at doses of 100, 200 and 400 mg/kg body weight, respectively. Groups 4, 5, 6 and 7 received 80 mg/kg of the *n*-hexane, ethyl acetate, *n*-butanol and aqueous residue fractions, respectively. Group 8 rats received atropine sulfate (batch EP304X) at 2.5 mg/kg body weight via the intraperitoneal route as the reference drug while group 9 animals were given compound 1 (2.5 mg/kg). The tenth group (negative control) received 5% ethanol/tween 80 in normal saline (1 ml/100 g body weight). After 50 min, all the animals were given 1 ml of vegetable charcoal meal (batch AJ0148) as a food tracer prepared at 10% in normal saline (0.9% sodium chloride). After an observation period of 40 min, each rat was sacrificed and dissected. The small intestine was removed and its total length was measured (cm). The movement of charcoal from the pylorus was equally measured (cm). The intestinal charcoal transit was expressed as a percentage of the distance moved by charcoal to the total length between the pylorus and the caecum.

### Shigella-Induced Diarrhea

The curative effects of plant extracts were evaluated on diarrhea induced by *Shigella flexneri* as described by Kamgang *et al*.[14] The inoculum was prepared at 9 × 10 ^8^ CFU/ml (McFarland 3 standard).Diarrhea provoked by *Shigella flexneri* in rats was further confirmed by the presence of blood in feces and white blood cells (WBCs) in fresh stool preparations.[15] The rats were randomly assigned to seven groups of eight animals each (four males and four females). Each animal was placed in a cage whose bottom was lined with a gauge that allowed the feces to pass through in order to minimize reinfection from fecal matter. The methanol extract and fractions were suspended in 5% tween 80 in 0.9% sodium chloride and orally administered to the rats at 100 and 80 mg/kg, respectively. Loperamide (2.5 mg/kg) was used to compare the plant extract activity alongside a negative control receiving only the vehicle.The evolution of treatment was assessed by examining the fecal Shigella load of stool, cultured on Salmonella–Shigella agar. Stool was collected on three consecutive days before the induction of diarrhea and five consecutive days after the induction. For this purpose, 0.5 g of feces was suspended in 4.5 ml sterile saline solution followed by serial dilutions. Then, 200 *μ*l of each dilution was cultured to count the number of Shigella colonies. Other recordings included animal weight variation and fecal frequency. At the end of the assay, all the animals were sacrificed and their blood, heart and liver were collected for the determination of total proteins[16] and blood cells. Sera triglycerides and creatinine levels were assayed using standard kits.

### Acute Oral Toxicity Evaluation

Eight-week-old Swiss mice (20–26 g), breed in the Animal House of the Department of Biochemistry (Faculty of Science, University of Dschang), were randomly selected and housed in standard plastic cages. They were starved for 18 h, but had access to tap water *ad libitum* prior to extract treatment. The 60 animals of the experiment were distributed into five groups of 12 animals each (six males and six females). Group 1 (saline control) did not receive plant extract while group 2–5 animals received 4, 8, 12 and 16 g/kg of the methanol extract, respectively. Mice were observed for mortality and behavioral changes for 48 h after treatment.[17]

### Statistical Analysis

Data were analyzed by one-way analysis of variance (ANOVA) and the results were expressed as mean ± standard deviation. The means were compared using the Waller–Duncan test at *P* ≤0.001.

## Results

### Phytochemical Analysis

**Figure 1:**
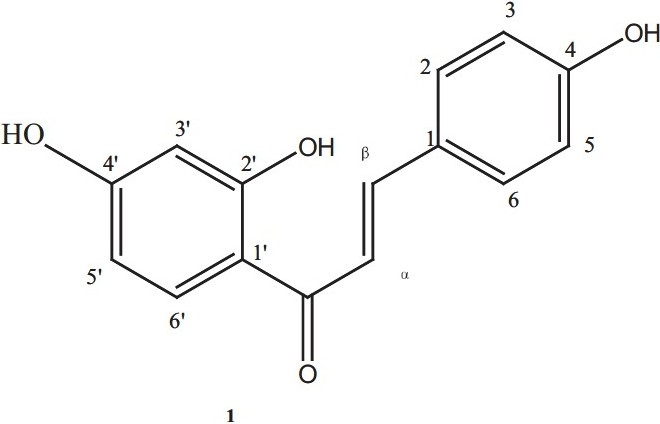
Chemical structure of isoliquiritigenin.

The qualitative analysis of the methanol extract and fractions of *T. madagascariense* revealed the presence of classes of compounds with potential antidiarrheal activities [Table 1]. Alkaloids, flavonoids, tannins, saponins and cardiac glycosides were detected.From spectroscopic analysis, compound 1was identified as isoliquiritigenin, with a molecular weight of 168.14672 and a melting point of 210–213°C [Figure 1].

**Table 1:** Phytochemical screening of *T. madagascariens* methanol extract and fractions

*Groups of chemical constituents*	*Methanol extract*	*Methanol extract subfractions*			
		*Hexane*	*Ethylacetate*	*n-butanol*	*Residue*
Alkaloids	+	-	-	-	+
Flavonoids	+	-	+	+	+
Coumarins	-	-	-	-	-
Polyphenols	+	-	+	+	+
Tannins	+	-	+	+	+
Saponins	+	-	+	+	+
Steroids	-	-	-	-	-
Triterpenes	-	-	-	-	-
Cardiac glycosides	+	-	-	-	+

### Effects of Test Samples on Fecal Characteristics in Castor Oil-Induced Diarrhea

The methanol extract, fractions and compound **1** from *T. madagascariense* stem bark significantly prolonged the time for diarrhea induction just like the standard drugs, loperamide and diphenoxylate HCl, when compared to the saline control rats. The latent periods were observed to increase with increase in drug dose. At 400 mg/kg, the purging indices and the inhibition percentages of defecation and wet stool for the methanol extract were comparable to those of the reference drugs at 2.5 mg/kg. Compound **1** (isoliquiritigenin) inhibited diarrheal droppings by 84.81%, similar to loperamide (72.65%) and diphenoxylate HCl (84.81%). The ethyl acetate fraction (80 mg/kg) was the most active fraction while the *n*-hexane fraction displayed the least antidiarrheal activity [Table 2].

**Table 2:** Effect of test materials from *T. madagascariense* at various doses on fecal characteristics in castor oil-induced diarrheal rats

*Test materials*	*Faecal characteristics*								
	*Dose (mg/kg)*	*% respondent*	*Latent period (min)*	*Total stool frequency*	*Purging indices*	*% inhibition of defecation*	*Frequency of wet stool*	*Water content (g)*	*Inhibition of wet stool (%)*
Saline control	0	100	60.80 ± 6.62	10.20 ± 1.31	16.77	0	7.90 ± 0.73	3.22 ± 0.55 0	0
Methanol extract#	100	100	78.60 ± 5.75*	6.50 ± 2.12*	8.26	36.27	3.50 ± 0.70*	2.05 ± 0.59*	55.69
	200	100	119.9 ± 6.90*	4.10 ± 0.56*	3.41	59.80	2.40 ± 0.84*	1.47 ± 0.21*	69.62
	400	80	270.50 ± 37.88*	3.25 ± 0.67*	0.96	68.13	1.50 ± 0.53*	1.07 ± 0.48*	81.01
n-hexane fraction	80	100	70.00 ± 5.35	8.10 ± 1.19*	11.57 20.58	5.00 ± 0.66*	2.88 ± 0.25*	36.70
Ethyl acetate fraction	80	60	186.50 ± 23.61*	3.70 ± 0.94*	1.19	63.72	2.16 ± 0.98*	1.03 ± 0.36*	72.65
n-butanol fraction	80	70	112.14 ± 15.68*	5.90 ± 1.59*	3.68	42.15	2.71 ± 1.11*	2.00 ± 0.60*	65.69
Aqueous residue	80	90	111.17 ± 19.22*	5.20 ± 1.03*	4.20	49.01	2.66 ± 0.86*	1.53 ± 0.37*	66.32
Diphenoxylate HCl	2.5	50	361.00 ± 29.66*	2.90 ± 0.70*	0.40	71.56	1.20 ± 0.44*	0.68 ± 0.20*	84.81
Loperamide	2.5	60	337.16 ± 26.53*	3.70 ± 0.67*	0.65	63.72	2.16 ± 0.75*	1.05 ± 0.25*	72.65
Isoliquiritigenina	2.5	50	179.40 ± 11.65*	3.80 ± 0.90*	1.05	62.74	1.20 ± 0.44*	0.88 ± 0.22*	84.81

Values are means ± SD; *n* = 10 in each group.,

* Significantly different from the saline control (*P* ≤ 0.001) Duncan test;

# The methanol extract of *T. madagascariens* was fractionated into the *n*-hexane, ethyl acetate, *n*-butanol and aqueous residues fractions; aIsoliquiritigenin, the isolated compound, was tested at the same dose as the reference drugs (diphenoxylate HCl and loperamide).

a Isoliquiritigenin, the isolated compound, was tested at the same dose as the reference drugs (diphenoxylate HCl and loperamide).

### Effect of Plant Extract on Enteropooling in Castor Oil-Induced Diarrhea in Rats

The volume and weight of intraluminal fluid in rats treated with the methanol extract of *T. madagascariense* significantly reduced, in a dose-dependent manner, almost to the same extent with respect to the untreated control rats [Table 3].

**Table 3:** Effect of test materials at various doses on mass and volume of intestinal fluids in rats

*Test materials*	*Dose (mg/kg)*	*Mass of intestinal fluid (×10^-2^ g/cm)*	*Volume of intestinal fluid (×10^-2^ ml/cm)*	*% inhibition of intraluminal fluid accumulation*
Saline control	0	3.54 ± 0.94	3.43 ± 0.31	0.00
Diphenoxylate HCl^a^	2.5	0.76 ± 0.14*	0.55 ± 0.21*	83.76
Loperamide	2.5	0.96 ± 0.19*	0.72 ± 0.32*	79.00
Methanol extract#	100	2.27 ± 0.21*	2.34 ± 0.23*	31.77
	200	1.68 ± 0.16*	1.69 ± 0.22*	50.72
	400	1.27 ± 0.14*	1.11 ± 0.19*	67.36

Values are means ± SD; *n* = 8 in each group;

* Significantly different from the saline control (*P* ≤ 0.001) Duncan test; The saline control was made 0% inhibition of intraluminal accumulation to reveal better the effects of the other test materials.;

# The methanol extract of *T. madagascariens*;

a The reference drugs were diphenoxylate HCl and loperamide.

### Effects of Plant Extracts on Gastrointestinal Motility

In the gastrointestinal motility test, the methanol extract at 100, 200 and 400 mg/kg body weight retarded the intestinal charcoal meal propulsion (%transit) in rats compared to the saline control for an observation period of 40 min. The *n*-hexane and the aqueous residue fractions at 80 mg/kg produced the least inhibitory effect on intestinal transit. Compound **1** and the other fractions inhibited intestinal motility almost to the same extent as atropine sulfate [Table 4].

**Table 4:** Effect of test substances on intestinal charcoal transit in rats

*Test substances*	*Dose (mg/kg)*	*Intestinal charcoal transit (%)*	*% inhibition of charcoal transit*
Saline control	0	73.07 ± 2.13	0
Atropine sulfate	2.5	31.06 ± 3.69*	57.49
Isoliquiritigenin	2.5	41.39 ± 5.54*	43.35
n-hexane fraction	80	56.33 ± 4.03*	22.90
Ethyl acetate fraction	80	39.33 ± 2.06*	46.17
n-butanol fraction	80	41.48 ± 2.89*	43.23
Aqueous residue fraction	80	54.33 ± 3.93*	25.64
Methanol extract	100	68.01 ± 1.77*	6.92
	200	59.94 ± 2.04*	17.96
	400	53.10 ± 3.23*	27.32

Values are means ± SD; *n* = 8 in each group;

* Significantly different from the saline control (*P* ≤ 0.001) Duncan test;

# The methanol extract of *T. madagascariens* was fractionated into the *n*-hexane, ethyl acetate, *n*-butanol and aqueous residues fractions;

a Isoliquiritigenin, the isolated compound, was tested at the same dose as the reference drugs (diphenoxylate HCl and loperamide).

### Effects of Plant Extracts on Fecal Frequency in Shigella-Induced Diarrhea

**Figure 2:**
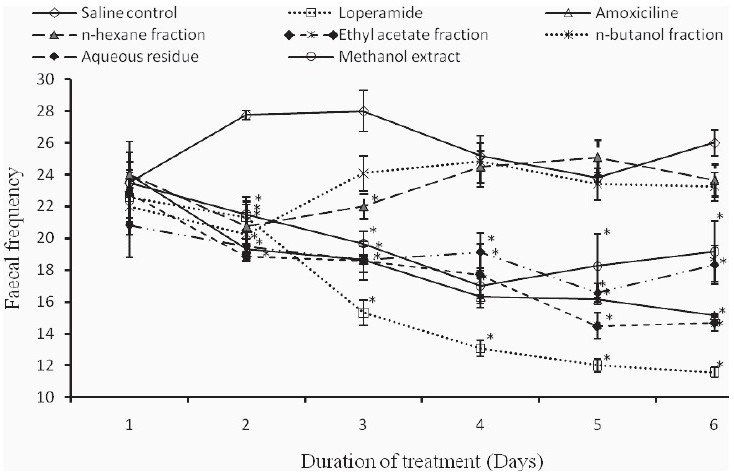
Variation of fecal frequency in Shigella-induced diarrhea in rats following treatment with test samples from *Trilepisium madagascariense*.

When diarrhea was induced by *Shigella flexneri*, the animals developed mild to severe diarrhea, characterized by the presence of thick liquid to bloody stool with occasional mucus. The fecal frequency of the saline-treated rats remained virtually high compared to those of the extract-treated animals [Figure 2].

### Effects of Treatment on Body Weight Gain in Shigella-Induced Diarrheic Rats

**Figure 3:**
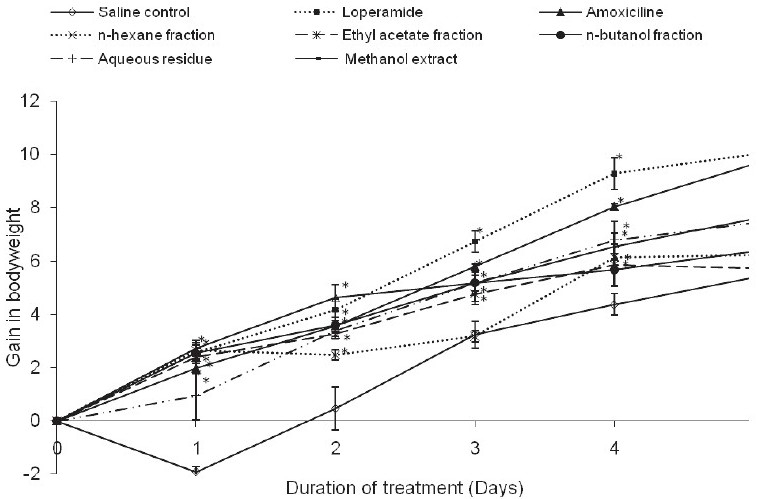
Evolution of gain in body weight in rats with Shigella diarrhea on treatment.

After Shigella-diarrhea induction, the gain in body weight was observed to increase with the duration of treatment. The saline control rats instead lost weight after day 1 of treatment. But, from day 2 up to day 6, the body weight gain was positive and was generally the least compared to the other test groups [Figure 3].

### Effects of Plant Extracts on the Faecal Shigella Load

**Figure 4:**
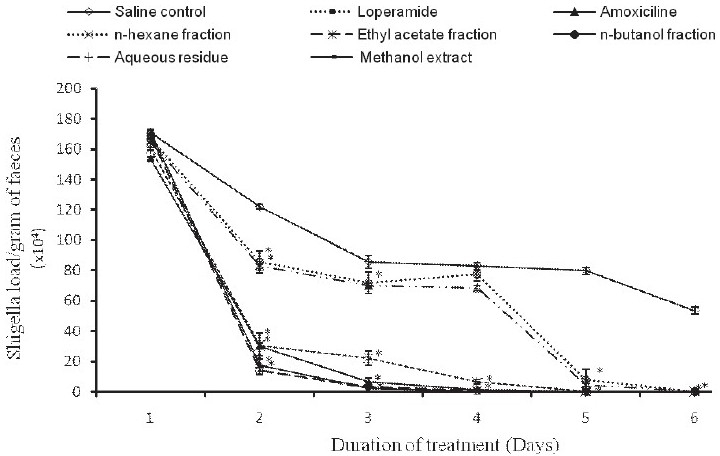
Evolution of faecal Shigella load in rats following treatment with test samples from *T. madagascariense*

It was observed that Shigella flexneri counts per gram of fecal matter in all the test animals decreased with the duration of treatment. By day 1 of treatment, a significant (*P* ≤0.001) reduction in fecal Shigella load was noted in all the test samples with respect to the control saline. *Shigella flexneri* was susceptible to the methanol extract and fractions of *T. madagascariense* stem bark. Complete eradication of shigellosis was obtained by the fourth day of treatment for the test samples, except for animals treated with the *n*-hexane and *n*-butanol fractions [Figure 4].

### Effects of Treatment on Some Hematological and Biochemical Parameters in Shigella-Induced Diarrhea in Rats

The red blood cells, WBCs and hematocrit levels of the animals at the end of the Shigella-induced diarrhea assay revealed a significant decrease (*P* ≤0.001) in the saline control, *n*-hexane and residue from those treated with the other test substances. The serum creatinine levels were significantly lower (*P* ≤0.001) in the plant extract and/or drug-treated animals compared to the saline control (40.75 ± 9.85 *μ*mol/L). The methanol extract (26.81 ± 5.65 *μ*mol/L), ethyl acetate fraction (24.66 ± 1.58 *μ*mol/L) and loperamide (18.23 ± 7.24 *μ*mol/L) were not significantly different. On the contrary, the serum triglycerides increased significantly in the extract-treated animals (ethyl acetate, 1.74 ± 0.35 mmol/L) from the saline control (0.68 ± 0.16 mmol/L). The protein levels recorded in the serum, heart and liver of the test animals equally displayed a significant increase (*P* ≤0.001) in the extract-treated groups with respect to those of the saline control [Table 5].

**Table 5:** Effect of treatment of Shigella diarrhea on some biochemical parameters in rats

*Treatment*	*Dose (mg/kg)*	*Biochemical parameters*							
		*Serum*						*Cardiac proteins (mg/g)*	*Hepatic proteins (mg/g)*
		*RBC (mm-3)*	*WBC (mm-3)*	*Hematocrit*	*Creatinine (μmol/L)*	*Triglyceride (mmol/L)*	*Proteins (mg/ml)*		
Saline control	0	55940 ± 2865	416 ± 75	29.90 ± 1.79	40.75 ± 9.85	0.68 ± 0.16	28.73 ± 3.54	34.34 ± 8.25	95.46 ± 19.20
Methanol extract	100	93,200 ± 9328*	643 ± 145*	36.80 ± 2.14*	26.81 ± 5.65*	1.50 ± 0.18*	40.47 ± 3.88*	57.7 ± 10.15*	177.18 ± 40.10*
n-hexane fraction	80	58,320 ± 3267	458 ± 59	30.00 ± 1.05	38.61 ± 7.49	1.21 ± 0.15*	31.06 ± 6.58	52.73 ± 14.19*	130.63 ± 28.61*
Ethyl acetate fraction	80	91,830 ± 12,726*	627 ± 94*	36.10 ± 1.79*	24.66 ± 1.58*	1.74 ± 0.35*	39.63 ± 1.04*	68.49 ± 10.76*	176.93 ± 16.05*
n-butanol fraction	80	83,090 ± 11,132*	583 ± 122*	32.10 ± 1.66*	28.96 ± 7.24*	1.29 ± 0.19*	30.78 ± 6.79	56.64 ± 5.59*	156.56 ± 47.68*
Aqueous residue fraction	80	56,430 ± 5228	242 ± 81	31.70 ± 1.88*	32.17 ± 7.15*	1.17 ± 0.39*	34.02 ± 5.64*	50.98 ± 8.98*	131.15 ± 38.37*
Loperamide	2.5	95,870 ± 11,586*	864 ± 87*	38.80 ± 3.04*	18.23 ± 7.24*	1.73 ± 0.19*	42.10 ± 2.77*	69.94 ± 10.86*	178.25 ± 8.22*

### Effects of Single Oral Dose of Methanol Extract Administered to Mice

From the acute toxicity study, all the mice administered the methanol extract survived. They appeared active and healthy, with no signs of abnormal behavior.

## Discussion

The action of castor oil as a diarrhea inductor has been largely studied, and its active component is ricinoleic acid, which produces an irritating and inflammatory action on the intestinal mucosa, leading to the release of prostaglandins. This condition increases the permeability of the mucosal cells and provokes changes in electrolyte transport thus causing diarrhea.[18]

The methanol extract significantly inhibited the castor oil-induced intestinal fluid accumulation (enteropooling) and weight of the intestinal content. The mechanism involved has been associated with dual effects of gastrointestinal motility as well as water and electrolyte transport (decreasing Na^+^ and K^+^ absorption) across the intestinal mucosa.[19] Thus, it is possible that the methanol extract of *T. madagascariense* reduced diarrhea by increasing reabsorption of electrolytes and water or by inhibiting induced intestinal accumulation of fluid just as the standard drugs like loperamide and diphenoxylate HCl.

In the evaluation of intestinal transit, atropine sulfate was used as the standard drug. Atropine is known to inhibit intestinal transit probably due to its anticholinergic effect.[20] The extracts and compound 1 (isoliquiritigenin) also appeared to act on all parts of the intestine. Thus, they reduced the intestinal propulsive movement in the charcoal meal-treated model. Isoliquiritigenin was first found in nature in 1953 from *Dahlia variabilis* (Compositae).[21] This compound has been reported as a potent relaxant.[22] Its activity against castor oil-induced diarrhea is being reported herein for the first time.

Antidiarrheal activity and antidysenteric properties of medicinal plants were found to be due to the presence of tannins, alkaloids, saponins, flavonoids, steroids and/or terpenoids.[2] These classes of compounds identified in our extract could be responsible for the biological activities of *T. madagascariense* extracts.

Protein synthesis was favored in animals that received post Shigella-diarrhea treatment. Diarrhea is known to reduce the rate of protein synthesis.[23] The low protein level and low blood cell counts observed in the saline control rats could be related to the losses in feces. A loss of blood and serum protein through inflamed intestine has been reported in children withshigellosis.[24] This situation can provoke a decrease in the body weight, as was observed in the saline-treated rats. Diarrhea, especially of infectious origin, is reported to provoke weight loss and retard growth rate.[25] The concomitant increase in serum creatinine levels and reduction in triglyceride levels have been reported in diarrheal cases.[23,25]

No deaths and no signs of abnormal behavior were observed in the mice treated with the methanol extract up to 16 g/kg bodyweight in the acute toxicity assay. This result suggests that adverse health effects following therapy with the methanol extract from *T. madagascariense* stem bark would not be expected, but this must be confirmed by further toxicological studies, including sub-acute and chronic toxicities.

## Conclusion

The results of our study, being reported for the first time, provide clear evidence that the methanol extract, fractions and isoliquiritigenin from *T. madagascariense* stem bark possess antidiarrheal activities and could be useful for the development of new antidiarrheal drugs. However, further pharmacological and toxicological studies will be necessary.
